# Supplementary material for: Patterns of Intron Gain and Loss in Fungi
Source: PLoS Biol. 2004 Nov 30;2(12):e422. doi: 10.1371/journal.pbio.0020422 (PMC532390; doi:10.1371/journal.pbio.0020422)
Supplement: Table S1 — Also available at http://genes.mit.edu/NielsenEtAl/. (4.3 MB ZIP). [file pbio.0020422.st001.zip › NielsenEtAl/html/1258.html]

AN2000.1.NCU05995.1.MG01282.2.FG08768.1


```
 CLUSTAL W (1.82) Multiple Sequence Alignments - Introns Inserted


Sequence 1: MG01282.2	305 aa
Sequence 2: FG08768.1	229 aa
Sequence 3: NCU05995.1	305 aa
Sequence 4: AN2000.1	323 aa
Alignment Length: 323 aa
Number Identitical Residues: 225 aa
Alignment Score (without introns) 9196


MG01282.2 	-------------~-----MQIFVKTL1TGKTITLEVESSDTIDNVKSKIQDKEGIPPDQ
NCU05995.1	-------------~-----MQIFVKTL1TGKTITLEVESSDTIDNVKQKIQDKEGIPPDQ
FG08768.1 	-------------~-------------~--------------------------------
AN2000.1  	MAAYKSIRVASVA2LVVDIMQIFVKTL1TGKTITLEVESSDTIDNVKTKIQDKEGIPPDQ
          	 :: .:   :: :    .  .   .:  :..: : . .::.: .. . . ..... ....

MG01282.2 	QRLIFAGKQLEDGRTLSDYNIQKESTLHLVLRLRGGMQIF~VKTLTGKTITLEVESSDTI
NCU05995.1	QRLIFAGKQLEDGRTLSDYNIQKESTLHLVLRLRGGMQIF~VKTLTGKTITLEVESSDTI
FG08768.1 	------------------------------------MQIF~VKTLTGKTITLEVESSDTI
AN2000.1  	QRLIFAGKQLEDGRTLSDYNIQKESTLHLVLRLRGGMQIF1VKTLTGKTITLEVESSDTI
          	.    :... ... : :. . ...::        ..**** *******************

MG01282.2 	DNVKSKIQDKEGIPPDQQRLIFAGKQLEDGRTLSDYNIQKESTLHLVLRLRGGMQIFVKT
NCU05995.1	DNVKQKIQDKEGIPPDQQRLIFAGKQLEDGRTLSDYNIQKESTLHLVLRLRGGMQIFVKT
FG08768.1 	DNVKSKIQDKEGIPPDQQRLIFAGKQLEDGRTLSDYNIQKESTLHLVLRLRGGMQIFVKT
AN2000.1  	DNVKTKIQDKEGIPPDQQRLIFAGKQLEDGRTLSDYNIQKESTLHLVLRLRGGMQIFVKT
          	**** *******************************************************

MG01282.2 	LTGKTITLEVESSDTIDNVKSKIQDKEGIPPDQQRLIFAGKQLEDGRTLSDYNIQKESTL
NCU05995.1	LTGKTITLEVESSDTIDNVKQKIQDKEGIPPDQQRLIFAGKQLEDGRTLSDYNIQKESTL
FG08768.1 	LTGKTITLEVESSDTIDNVKSKIQDKEGIPPDQQRLIFAGKQLEDGRTLSDYNIQKESTL
AN2000.1  	LTGKTITLEVESSDTIDNVKSKIQDKEGIPPDQQRLIFAGKQLEDGRTLSDYNIQKESTL
          	********************.***************************************

MG01282.2 	HLVLRLRGGMQIF~VKTLTGKTITLEVESSDTIDNVKSKIQDKEGIPPDQQRLIFAGKQL
NCU05995.1	HLVLRLRGGMQIF~VKTLTGKTITLEVESSDTIDNVKQKIQDKEGIPPDQQRLIFAGKQL
FG08768.1 	HLVLRLRGGMQIF1VKTLTGKTITLEVESSDTIDNVKSKIQDKEGIPPDQQRLIFAGKQL
AN2000.1  	HLVLRLRGGMQIF~VKTLTGKTITLEVESSDTIDNVKTKIQDKEGIPPDQQRLIFAGKQL
          	************* *********************** **********************

MG01282.2 	EDGRTLSDYNIQKESTLHLVLRLRGGQ
NCU05995.1	EDGRTLSDYNIQKESTLHLVLRLRGGQ
FG08768.1 	EDGRTLSDYNIQKESTLHLVLRLRGGQ
AN2000.1  	EDGRTLSDYNIQKESTLHLVLRLRGGN
          	**************************:
```
